# Turtle Tuberculin

**Published:** 1914-04

**Authors:** 


### Turtle Tuberculin

It is natural that, after the enthusiasm and prolific press reports of the Friedmann treatment, there should follow a period of apathy. But the main consideration is not loss of novelty, not disgust with commercialism, not even the fact that the Friedmann treatment failed to make good. The essential thing is whether the hint received from Friedmann will lead to practical results. Piorkowski began studying chelonic tubercle bacilli in 1903. He has recently claimed to have produced an efficient chelonic tuberculin. Friedmann contended, without claim of originality, and with considerable authority, that a non-virulent, living bacillus, and not an extract or dead bacillus, must be looked to for hope of efficient immunization. This general claim has been denied. Drs. J. W. Beattie and E. E. Meyers (*N. Y. Med. Jour.*, September 13, 1913) report very encouraging results from turtle tuberculin. At any rate, it is worth while to continue searching for some specific treatment for tuberculosis; to thresh out the question between immunization by non-virulent strains and by chemic products of the bacillus; and, in particular, to settle the availability of chelonic strains, among others, in either of these directions.

### FAILURE NOT NECESSARILY AN INDICATION OF WORTHLESSNESS

This title was not taken to point a hopeful moral to discouraged individuals though, in passing, we may suggest that success consists largely in hanging on. It is said that the late D. Hayes Agnew made a failure of medicine, went into the coal business and later returned to practice and, as is well known, was signally successful in every sense. Very few men, retiring prematurely from medical practice or any other line of work, can claim to have made a success. On the other hand, almost every man who has persisted in any line of work, to the end, can fairly claim to have been measurably successful. If we stop to think about it, success means doing better than 95 per cent. of our competitors, and not more than 5 per cent. of us can do this, though a considerably larger percentage claim to have accomplished this desirable result.

What we started to say was that, under present conditions, any new discovery or "method" or "system," medical or otherwise, that attracts much attention, either explicitly or implicitly, claims a degree of efficiency far beyond the average. Only a small percentage can be highly successful. Yet the fact that a discovery or method fails to satisfy the hopes aroused at the outset, does not, by any means, prove that it is worthless. Morphine, digitalis, salicylates, or a dozen other drugs held in the highest esteem by 99 per cent. of the profession would prove failures if heralded as new discoveries and as sure cures for the conditions to which they are almost unanimously considered applicable. We hold no brief for the Friedmann treatment but, merely to illustrate the point, it may be said that any one of these justly esteemed drugs would have failed if tested, for a few months, under conditions fairly comparable with those to which the Friedmann vaccine was subjected. Do not misunderstand this statement to mean that we are courting unpopularity by supporting Friedmann at this late day. But let us not forget that the only test of worth, except in very occasional and exceptional revolutionary discoveries, is that obtained after several years by a great many independent investigators, and that we must consider not the results in unfavorable cases, not phenomenal cures, but moderate and even temporary mitigation of evils.

